# DNA Instability at Chromosomal Fragile Sites in Cancer

**DOI:** 10.2174/138920210791616699

**Published:** 2010-08

**Authors:** Laura W Dillon, Allison A Burrow, Yuh-Hwa Wang

**Affiliations:** Department of Biochemistry, Wake Forest University School of Medicine, Medical Center Boulevard, Winston-Salem, NC 27157-1016, USA

**Keywords:** ATR checkpoint pathway, cancer-specific chromosomal translocation, DNA secondary structure, environmental mutagen, fragile site, RET/PTC rearrangement, stalled replication fork.

## Abstract

Human chromosomal fragile sites are specific genomic regions which exhibit gaps or breaks on metaphase chromosomes following conditions of partial replication stress. Fragile sites often coincide with genes that are frequently rearranged or deleted in human cancers, with over half of cancer-specific translocations containing breakpoints within fragile sites. But until recently, little direct evidence existed linking fragile site breakage to the formation of cancer-causing chromosomal aberrations. Studies have revealed that DNA breakage at fragile sites can induce formation of *RET/PTC* rearrangements, and deletions within the *FHIT* gene, resembling those observed in human tumors. These findings demonstrate the important role of fragile sites in cancer development, suggesting that a better understanding of the molecular basis of fragile site instability is crucial to insights in carcinogenesis. It is hypothesized that under conditions of replication stress, stable secondary structures form at fragile sites and stall replication fork progress, ultimately resulting in DNA breaks. A recent study examining an FRA16B fragment confirmed the formation of secondary structure and DNA polymerase stalling within this sequence *in vitro*, as well as reduced replication efficiency and increased instability in human cells. Polymerase stalling during synthesis of FRA16D has also been demonstrated. The ATR DNA damage checkpoint pathway plays a critical role in maintaining stability at fragile sites. Recent findings have confirmed binding of the ATR protein to three regions of FRA3B under conditions of mild replication stress. This review will discuss recent advances made in understanding the role and mechanism of fragile sites in cancer development.

## INTRODUCTION

Genomic instability is a common cause of chromosomal aberrations in many types of tumor cells. Cells can acquire DNA damage through various extrinsic or intrinsic factors. DNA damage commonly arises following exposure to exogenous factors such as UV radiation, ionizing radiation, chemotherapy, and endogenous factors such as reactive oxygen species [1]. Chromosomal fragile sites, which are especially susceptible to DNA breakage, have also been suggested as contributory to the formation of cancer-specific chromosomal aberrations [2]. 

Chromosomal fragile sites are defined as regions of the genome which exhibit gaps or breaks on metaphase chromosomes under conditions of partial replication stress [3]. Many genes identified as tumor suppressors or oncogenes are located at or within fragile sites [4]. The deletion of tumor suppressors and the amplification of oncogenes are frequently consequences of breakage at these sites. Studies have also demonstrated a significant association between sites of breakage in cancer-specific chromosomal rearrangements and the location of fragile sites [5-7]. Several oncogenic viruses target and preferentially integrate at these chromosomal regions [4]. Based on this information, it has been proposed that fragile sites may directly contribute to cancer development, although their direct role remains unclear. In a recent study by Gandhi *et al.*, DNA breakage at fragile sites directly contributed to the formation of a cancer-specific chromosomal translocation found in human papillary thyroid carcinoma [8]. 

Fragile sites are divided into two major classes, based on their frequency in the population, and are further divided according to their mode of induction in cultured cells. Common fragile sites have been observed in all individuals and are therefore believed to represent a normal component of chromosome architecture [9]. Most common fragile sites are induced by low doses of aphidicolin (APH), an inhibitor of DNA polymerases α, δ and ε [10, 11]. Other common fragile sites can occur following treatment with bromodeoxyuridine (BrdU) or 5-azacytidine (5-aza). In contrast, rare fragile sites are found in less than 5% of the population and are inherited in a Mendelian manner [12, 13]. Most of these sites are expressed under folate-deficient conditions, whereas others are induced by chemicals that bind AT-rich DNA, such as distamycin-A or berenil. Recently, Mrasek *et al.* found that APH can induce all types of common and rare fragile sites, suggesting that their expression is less dependent on their currently defined mode of induction, and instead, a classification of fragile sites based on their frequency is more appropriate [14]. In addition, various dietary and environmental factors can significantly increase fragile site breakage, including caffeine, ethanol, pesticides, and cigarette smoke [3].

While the molecular basis remains elusive, several factors may contribute to fragile site breakage. Although a consensus sequence has not yet been identified among all fragile sites, all common fragile DNAs examined to date are comprised of AT-rich flexibility islands, with the potential of forming secondary structures that are much more stable than other genomic regions [15, 16]. These regions can extend over megabases of DNA, with gaps or breaks occurring throughout [17]. Fragility at rare sites is attributed to the expansion of either a CGG repeat or an AT-rich minisatellite, which can also form stable secondary structures. All fragile sites studied so far are late-replicating regions of the genome, and their replication can be further delayed in the presence of induction agents, with some fragile site alleles remaining unreplicated in late G_2 _[18-22]. In addition, the ATR (Ataxia-telangiectasia and Rad3 Related)-dependent DNA damage checkpoint pathway is crucial for fragile site maintenance, since a deficiency of proteins including ATR [23, 24] and its downstream targets, such as BRCA1 [25] and CHK1 [26], result in a dramatic increase in fragile site breakage. Therefore, it is hypothesized that at fragile sites, under conditions of replication stress, the replicative polymerases may uncouple from the helicase, resulting in long regions of single-stranded DNA and thus promoting the formation of stable secondary structures. Consequently, these structures may stall replication fork progression, triggering an ATR pathway response. Defective ATR pathway signaling could result in DNA breakage at these sites, and thus lead to cancer-specific chromosomal aberrations [6].

Here, we will review recent advances made in understanding the role and mechanism of fragile sites in cancer development. We will discuss the importance of fragile sites, direct evidence of their involvement in cancer, sequence characteristics of fragile sites that contribute to their instability, and the role of the ATR-dependent DNA damage checkpoint pathway in their breakage.

## IMPORTANCE OF FRAGILE SITES IN CANCER

Fragile sites are normally stable in cultured cells. However, these regions are highly susceptible to chromosomal deletions, rearrangements, and sister chromatid exchanges following their induction with replication inhibitors [27, 28]. Most rare fragile sites can be induced by removal of folic acid or treatment with fluorodeoxyuridine (FUdR), an inhibitor of folate metabolism. Other rare sites are expressed upon treatment with minor groove binders, such as distamycin-A or berenil, as well as BrdU, a nucleoside analog of thymidine that is incorporated into newly synthesized DNA. BrdU is also an inducer of several common fragile sites, along with 5-aza, whereas most sites are induced by APH, an inhibitor of DNA polymerases α, δ and ε. Common fragile sites can also be induced by various environmental agents and chemicals, including caffeine, ethanol, and cigarette smoke (Table**1**).

Caffeine, an inhibitor of phosphoinositide 3-kinase related kinases, including ATR and ATM (Ataxia-telangiectasia mutated), significantly increases common fragile site breakage in conjunction with FUdR and APH [5, 29]. Ethanol and APH also significantly increases fragile site breakage [30]. Interestingly, cells from chronic alcohol users show a significantly higher frequency of fragile site and chromosomal breakage compared to control individuals, suggesting long term alcohol use alone can induce fragile site expression [31]. Hypoxic conditions induced fragile site breakage in GMA32 Chinese hamster cells, with or without the addition of other fragile site-inducing chemicals [32]. Kao-Shan *et al.* found that peripheral blood lymphocytes from cigarette smokers show significantly greater fragile site breakage compared to non-smokers [33]. Stein *et al.* found that treatment of peripheral blood lymphocytes with low-doses of APH results in increased fragile site breakage in active smokers compared to non-smokers and patients with small cell lung cancer who stopped smoking [34]. These results suggest that active exposure to cigarette smoke increases the potential of breakage at fragile sites, and that this risk is reversible. 

Exposure to pesticides also results in an increased susceptibility to fragile site breakage. Blood lymphocytes from pesticide sprayers and flower collectors working in greenhouses show greater fragile site breakage than normal individuals following treatment with APH, with these results being reproducible a year later [35, 36]. An association between pesticide exposure and an increased risk of hematopoietic tumors has been observed [37, 38]. The APH-induced damage was enhanced at fragile sites containing breakpoints involved in leukemias and non-Hodgkin’s lymphoma, supporting a role for pesticide-associated fragile site breakage in development of these cancers. Like cigarette smoke exposure, the effect of pesticide exposure on fragile site breakage is also transient. APH-induced fragile site breakage in blood cells from farmers increased significantly post-exposure to organophosphate-based pesticides compared to pre-exposure samples, where the farmers had not been exposed to pesticides for at least one month [39]. 

Several mutagens and carcinogens can also induce fragile site breakage. Yunis *et al.* screened various mutagens for their ability to induce fragile site breakage [40]. The mutagens found to induce breakage were diverse and included benzene, a component found in cigarette smoke and gasoline fumes; carbon tetrachloride, formerly widely used in refrigerants and pesticides; diethylnitrosamine, found in cigarette smoke, pesticides, cured meat, and whiskey; and dimethyl sulfate, used in the manufacturing of dyes, drugs, perfumes, and pesticides. Several chemotherapeutic agents were also identified, including actinomycin D, bleomycin, busulfan, chlorambucil, cytosine arabinoside, 5-aza, FUdR, and methotrexate. The action of these mutagens was enhanced 3- to 8-fold with the addition of caffeine.

Peripheral blood lymphocytes from hypertensive patients taking atenolol, a common β-blocker, have more frequent chromatid and chromosome breaks than normal individuals, and these breaks are preferentially located at fragile sites [41]. While antihypertensive drugs have been examined for genotoxicity and carcinogenicity, a recent review suggests that studies to date may lack sufficient evidence to evaluate the potential risks of these drugs in humans [42]. One genotoxicity study of atenolol revealed a statistically significant increase in the number of micronuclei in patents taking atenolol compared to control [43]. Combined, these data suggest that more long-term research needs to be performed to assess the risk of these drugs on carcinogenesis, and that fragile site breakage may be involved in the carcinogenesis process.

Variability of fragile site breakage has been observed within individuals [44]. This phenomenon may reflect exposure to such mutagens/carcinogens, with high levels being associated with cancer patients [45]. Tumor cells also demonstrate an association between sites of breakage in recurrent chromosome abnormalities and the position of fragile sites. The two most highly breakable fragile sites, FRA3B and FRA16D, lie within the tumor suppressor genes *FHIT* and *WWOX*, respectively. The *FHIT* gene is often involved in deletions which map specifically to the fragile site region in various types of cancer [46-51], and deletion of *WWOX* is also frequently observed in tumor cells [52-57]. It has been proposed that fragile sites may also play a role in the breakage-fusion-bridge model of gene amplification [58, 59], since proto-oncogenes such as *MYC* [60] and *MET *[61] are located at fragile sites FRA8C and FRA7G, respectively. In addition to environmental mutagens, several oncogenic viruses including human papilloma virus [62], Hepatitis B [63], and Epstein-Barr [64], have been shown to target and preferentially integrate at fragile sites. 

While strong correlations exist between fragile site locations and sites of breakage in other chromosome rearrangements, such as translocations, until recently there was no systematic demonstration of the location of fragile sites relative to all reported translocations in tumor cells. After the examination of all known cancer-specific recurrent translocations, it was determined that over half (52%) of translocation breakpoints in participating gene sets correspond to fragile site positions [65]. Furthermore, 65% of the break-points were located within common fragile sites, as opposed to rare sites, and most cancers associated with the translocations examined had little or no genetic component. These results suggest that exposure to fragile site-inducing chemicals may confer a risk for the formation of cancer-specific rearrangements. This study focused on simple translocations involving two genes, or deletions which lead to fusion transcripts, and excluded single gene deletions (the most common aberrations associated with fragile sites) and chromosomal insertions or more complex translocations involving multiple genes. Therefore, the association between translocation breakpoints and fragile site locations revealed in this study is likely to be an underestimation.

Because the evidence strongly supports a link between fragile sites and cancer development, it is essential to investigate the mechanism of fragile site breakage, and to examine the consequences of breakage at these sites in order to demonstrate a causative role in tumorigenesis. Also, it will be important to identify factors that contribute to chromosomal fragility, such as DNA sequence, proteins and environmental/dietary agents, since fragile sites can be induced by a variety of environmental and chemical agents.

## DIRECT EVIDENCE OF FRAGILE SITE INVOLVEMENT IN CANCER DEVELOPMENT

Despite a long-established connection between fragile sites and the formation of cancer-specific chromosomal aberrations in many different studies [66], no studies have demonstrated direct evidence of fragile site breakage leading to cancer-causing chromosomal aberrations. Most experiments testing fragile site induction through exposure to chemicals examined DNA breakage on the cytogenetic level. However, two recent studies examining fragile site breakage at the nucleotide level, have revealed that DNA breakage at fragile sites can lead to the formation of *RET/PTC* rearrangements [8], as well as deletions within the *FHIT* gene [67], resembling those observed in human tumors.

FRA3B, the most frequently expressed fragile site in the genome, is located within the tumor supressor gene *FHIT *[68]. Deletions within *FHIT* have been associated with various human cancers including breast, lung, cervical, and esophageal [66]. Durkin *et al.* observed submicroscopic deletions within *FHIT*/FRA3B following treatment of human-mouse chromosome 3 somatic hybrid cells with low doses of APH [67]. The APH-induced deletions spanned ~200-600 Kb within *FHIT* and were centered on exon 5, within the breakpoint cluster of FRA3B. The location and size of APH-induced *FHIT* deletions were consistent with those observed in esophageal cancer cell lines, small-cell and non-small cell lung carcinomas, and breast cancers. Sequence analysis of the APH-induced deletion breakpoints showed no sequence homology, suggesting nonhomologous end-joining (NHEJ)-mediated repair. Interestingly, chromosomes from clones containing APH-induced FRA3B deletions exhibited a significant decrease in FRA3B breakage following additional APH treatment. While these studies show directly that fragile site breakage can lead to mutations like those seen in human tumors, these experiments were carried out in mouse hybrid cells, which may not respond similarly to human cells.

Genes participating in the two major types of *RET/PTC* rearrangements, *RET/PTC1* and *RET/PTC3*, are all located within known common fragile sites [65]. *RET/PTC* rearrangements, a common cause of papillary thyroid carcinoma, result in the fusion of the *RET* tyrosine kinase domain to the 5’ portion of various constitutively expressed genes [69]. *RET *(*re*arranged during *t*ransfection) encodes for a cell membrane receptor tyrosine kinase protein whose ligands belong to the glial cell line-neurotropic factor (GDNF) family [70]. In the thyroid gland, RET is highly expressed in neural crest derived C-cells but not in follicular cells, where it can be activated through the formation of *RET/PTC *rearrangements [69]. In *RET/PTC1*,* RET* is rearranged with *CCDC6*, while in *RET/PTC3* is rearranged with *NCOA4* [71]. Both *CCDC6* and *NCOA4* are located closer than expected to *RET* in interphase nuclei of normal human thyroid cells, potentiating rearrangement formation [72, 73]. *RET* and *NCOA4* are both located within the APH-induced common fragile site FRA10G, and *CCDC6* is located within the BrdU-induced common fragile site FRA10C. Recently, Gandhi *et al.* found that exposure of human thyroid epithelial cells to the fragile site-inducing chemicals APH, BrdU, and 2-AP results in the formation of *RET/PTC1* rearrangements like those observed in patients [8]. DNA breakage was observed within *RET*, *CCDC6*, and to a lesser extent *NCOA4* using fluorescence *in situ *hybridization, following exposure to fragile site-inducing chemicals, consistent with the mode of induction for the fragile site where each gene is located. 

APH-induced DNA breakage within *RET* was identified to be located within intron 11 [8], the major breakpoint cluster region within patients [74]. Interestingly, these breakpoints were located within 2-15 basepairs of breakpoints identified from human papillary thyroid carcinomas containing *RET/PTC* rearrangements. The breakpoints isolated in patient samples were identified post-rearrangement, while the breakpoints induced by APH were identified pre-rearrangement. In most patient tumors, small insertions or deletions ranging from 1-18 nucleotides surround the fusion points, suggesting a possible break repair mechanism after the initial breaks that form the rearrangement. Schwartz *et al.* found that downregulation of Rad51, DNA-PKcs, and Ligase IV, key components of the homologous recombination (HR) and NHEJ repair pathways, significantly increases fragile site breakage with APH treatment, and that γH2AX and phosphorylated DNA-PKcs foci were located at expressed fragile sites [75]. Together, these data suggest a role for both the HR and NHEJ repair pathways in the repair of fragile site breakage. However, more research is needed to elucidate the contribution of these pathways in the formation of *RET/PTC* rearrangements as a result of fragile site breakage.

*	RET/PTC* rearrangements are commonly associated with radiation exposure, especially in children. However, most adult tumors containing *RET/PTC* rearrangements are sporadic and patients lack a history of radiation exposure [76]. Most importantly, the observation that fragile site-inducing chemicals can cause the formation of *RET/PTC1* rearrangements, and significant breakage at *RET* and *CCDC6* but not *NCOA4 *[8], suggests a role for fragile sites in the formation of sporadic *RET/PTC1* tumors. Interestingly, an increasing prevalence of *RET/PTC1* over *RET/PTC3* rearrangements has been observed in sporadic papillary thyroid carcinomas [77], which is consistent with the experimental data and further supports fragile site involvement in sporadic papillary thyroid carcinomas. Overall, these experiments show direct involvement of fragile sites in the formation of cancer-causing chromosomal translocations within human cells.

According to the National Cancer Institute, thyroid cancer is now the fastest growing cancer among both men and women, increasing at a rate of 6.5 percent a year from 1997-2006 [78]. Of all types of thyroid cancer, papillary thyroid carcinoma is the only subtype in which the rate of incidence increased consistently regardless of race or ethnicity [79]. Interestingly, in a study examining cancer incidence in U.S. Air Force active duty personnel between 1989-2002, thyroid cancer was the third most frequent invasive cancer in women and the fifth most frequent in men [80]. Compared to the U.S. general population, thyroid cancer in women was over four times more prevalent in the U.S. Air Force. More importantly, overall cancer incidence in the U.S. Air Force were significantly reduced compared to the general population, suggesting that the increase in thyroid cancer may partly be the result of occupational exposure. Several components of unique occupational conditions for active duty Air Force personnel, including jet fuel, napalm, and high altitudes, have been shown to induce fragile site breakage. While the cause of the increased thyroid cancer incidence is unknown, exposure to various environmental and dietary fragile site-inducing chemicals and conditions is a potential causal factor.

## DNA SECONDARY STRUCTURE AND REPLICATION FORK STALLING AT FRAGILE SITES

Understanding the molecular basis of fragile site breakage is critical for dissecting the role of fragile sites in cancer. Several intrinsic factors may contribute to their expression. Replication timing experiments have demonstrated that all fragile sites examined so far, including FRA1H [22], FRA2G [22], FRA3B [19], FRA7H [20], FRA10B [21], FRA16B [21], and FRAXA [18] exhibit late replication, which can be further delayed by the addition of replication inhibitors, with some fragile site alleles remaining unreplicated in the late G_2_ phase [19, 20]. Although there is no consensus sequence among fragile sites, most fragile DNAs studied to date can form highly stable secondary structures [15, 16]. Evidence for secondary structure formation at fragile sites has largely been generated by the Mfold program [81], which predicts secondary structure formation of a single-stranded DNA. The first evidence of fragile DNA forming a secondary structure *in vitro* came from the ability of the CGG repeat, which underlies the basis of fragility at rare, folate-sensitive fragile sites, to form both quadruplex [82] and hairpin structures [83]. Furthermore, the formation of such structures by the CGG repeat presents a significant block to replication both *in vitro* [84] and *in vivo* [85]. Additionally, a polymorphic AT-rich sequence within the common fragile site FRA16D is predicted to form a cruciform that can block replication and increase chromosomal instability in yeast [86]. A recent study examining the rare fragile site FRA16B demonstrated the ability of an AT-rich site, which comprises the majority of fragile sites, to form a secondary structure *in vitro *[87].

Several studies have demonstrated a significant effect of *cis*-acting factors, including replication orientation and distance relative to the origin on the instability of fragile site DNA sequences. This effect may be due to the ability of an unstable DNA to form a stable secondary structure, and/or location of an alternative DNA structure within a region that maintains single-strandedness during replication, such as the Okazaki initiation zone (OIZ), which could promote the formation of such structures. Reports examining replication of expanded trinucleotide repeats in mammalian cells, such as CTG [88], GAA [89], and CGG [90], indicate that when the more structure-prone strand serves as the lagging strand template (a situation dictated by replication orientation), mutation events increase. The types of mutations (i.e. insertions and deletions) also differed based on the orientation of the DNA. When both strands can form an alternative structure (as with FRA16B), a similar orientation effect is also seen, likely due to the difference in the structures, as evidenced by the different electrophoretic mobilities produced by the two strands in native polyacrylamide gels [87]. The mutation rate for the clone in which the more stable structure served as the lagging strand template was statistically significant higher than that of the clone in opposite orientation [87]. These studies also revealed a significant effect on the distance from the repeat to the replication origin. Placing a repetitive fragment of DNA close to the origin of replication would cause the unstable sequence to occupy the first OIZ, potentially out-competing the binding of RPA protein. Repeats located further away from the origin would occupy different OIZs, and could be trumped by the initial binding of RPA in the first OIZ, which would propagate into the second, third, and so on [87]. In addition to mutation rate, replication orientation and distance relative to the origin also significantly affect replication efficiency of CGG and FRA16B-containing constructs. Edamura *et al.* found that constructs with expanded CGG repeats located further from the origin of replication replicated less efficiently compared to constructs with the same orientation located closer to the origin; plasmids in which CGG repeats served as the lagging strand template demonstrated a higher replication rate relative to plasmids in which CCG repeats served as the lagging strand template [90]. In a study of FRA16B replication in human cells, Burrow *et al.* found that various FRA16B-containing constructs replicated less efficiently compared to a control without fragile DNA, and that both orientation and distance from the origin significantly affected its replication rate [87].

A decrease in replication rate by a stalled fork could be responsible for the late replication observed at fragile sites. To date, synthesis of fragile site DNA has been investigated using primer extension assays both *in vitro* and in human cell-free extracts, and analyzing synthesis intermediates isolated for 2D gel electrophoresis in yeast. Examination of replication intermediates from cells containing AT-rich sequences within common fragile site FRA16D in *S. cerevisiae* showed site-specific replication fork stalling, depending on the length of the AT repeat [86]. Furthermore, correlation with secondary structure predictions suggests that the structure formed by this repeat is responsible for the fork stalling. Using an *in vitro* primer extension assay, synthesis of the same fragile site by human replicative polymerases δ and α confirmed polymerase stalling at sites predicted to form inhibitory DNA structures, which was alleviated by the addition of WRN protein [91]. The same study examined primer extension using HeLa cell-free extracts, and obtained similar results. Interestingly, FRA16D regions with increased DNA flexibility and accompanying high A/T content were not sufficient to inhibit DNA synthesis, but sequences with the propensity to form secondary structures were significantly more inhibitory to replication. Fragile site FRA16B exhibits characteristics of both common and rare sites, and showed strong polymerase pause sites specifically within the fragile DNA sequence *in vitro*, likely due to its ability to form an alternative DNA structure [87].

These data strongly suggest that the secondary-structure forming ability of fragile site DNAs greatly contributes to their instability by inhibiting replication. Even without replication stress, fragile sites show replication blockage during synthesis. While fragile site instability primarily arises from DNA polymerase stalling, visualization of FRA16B replication fork constructs by electron microscopy showed a high propensity for stalled forks to spontaneously regress during synthesis, a previously unidentified mechanism of instability at these sites [87]. In addition, measurements of the non-regressed FRA16B tracts confirmed synthesis products that were much shorter than predicted, consistent with the deletion mutants produced from replication of FRA16B in HEK293T cells. These results suggest yet another mechanism of fragile site instability in which polymerase bypass may occur at regions of secondary structure formation. Based on these results, fragile DNAs likely contain intrinsic features that make them difficult to replicate, and in the presence of replication stress, which can be produced by the addition of chemicals such as aphidicolin, replication is further delayed. The addition of aphidicolin or other fragile site-inducing chemicals will likely create an accumulation of long, single-stranded DNA regions caused by the functional uncoupling of replicative DNA polymerase and helicase activities. These regions are then highly prone to forming stable secondary structures which could further stall progression of the replication fork, triggering activation of ATR and its downstream products. A small fraction of fragile sites escapes the replication checkpoint, which would lead to breakage at fragile sites.

## REGULATION OF COMMON FRAGILE SITE STABILITY BY THE ATR PATHWAY

ATR kinase is a DNA damage sensor protein that has a major role in regulating stability at common fragile sites. ATR works with downstream target proteins to respond to stalled and collapsed replication forks, resulting in a block in further replication and mitosis progression and the promotion of DNA repair, recombination, or apoptosis [92, 93]. The loss of functional ATR in cells results in a defective DNA damage response to agents which block replication fork progression, including APH and hydroxyurea [94-96], and conditions of hypoxia [97]. Casper *et al.* found that cells deficient in ATR, but not ATM, display up to a 20-fold increase in fragile site breakage following treatment with low doses of APH compared to control cells [23]. Also, a deficiency in ATR alone is enough to induce fragile site breakage in cells without treatment with replication inhibitors. Cells from patients with Seckel syndrome, who express low levels of ATR protein due to a hypomorphic mutation in the *ATR* gene, exhibit an increase in chromosomal breakage at common fragile sites compared to unaffected individuals [24]. Furthermore, mice hypomorphic for ATR also display an increase in common fragile site breakage and a significant delay in checkpoint induction [98]. While the loss of ATM alone does not cause increased common fragile site breakage [23], it is involved in maintaining fragile site stability in the absence of ATR. Ozeri-Galai *et al.* found that a loss of both ATR and ATM significantly increases APH-induced common fragile site breakage compared to the loss of ATR alone. Also, ATM is activated and forms nuclear foci with γH2AX following treatment with low doses of APH [99]. These findings indicate that ATR is the major pathway responsible for maintaining fragile site stability, but that ATM also plays a secondary role, perhaps through a downstream response to double strand breaks that form as a result of ATR deficiency.

Other downstream targets of the ATR-mediated pathway involved in maintaining fragile site stability include BRCA1 [25], CHK1 [26], SMC1 [100], FANCD2 [101], HUS1 [102], WRN [103], and Claspin [104] (Table **2**). BRCA1 is a primary target of both ATR and ATM phosphorylation in response to DNA damage. Cells lacking BRCA1 show significantly more fragile site breakage after treatment with APH compared to control cells [25]. Also, cells expressing mutant BRCA1 exhibit elevated levels of fragile site breakage but lack the G_2_/M checkpoint, suggesting BRCA1 regulates fragile site stability through its role at this checkpoint.

CHK1 kinase is the major downstream target of ATR and serves as the central regulator of the ATR checkpoint pathway. Loss of CHK1, but not the ATM regulated CHK2, in cells was found to result in a significant increase in fragile site breakage after treatment with APH [26]. Also, it was found that both ATR and ATM phosphorylate CHK1 following treatment with low doses of APH [99]. These data suggest that the role of ATM in fragile site maintenance may be to activate the ATR pathway through phosphorylation of CHK1, when ATR is missing or fails to properly respond to damage. HUS1 is a member of the PCNA-related 9-1-1 complex which promotes the phosphorylation of ATR substrates like CHK1 and helps aid in DNA repair through association with multiple factors. A significant increase in DNA breakage at common fragile sites was observed after inactivation of HUS1 [102]. SMC1 is a chromosomal structural maintenance protein that belongs to the cohesin complex, which is necessary for sister chromatid cohesion and DNA repair and acts to hold DNA strands in place. After treatment with APH, cells exhibit an ATR-dependent, ATM-independent, phosphorylation of SMC1 and increased fragile site breakage after SMC1 inhibition [100]. Claspin is another member of the ATR pathway that is required for ATR-mediated phosphorylation of CHK1 in response to replication stress. Inhibition of claspin expression increases fragile site expression, with or without APH treatment [104].

Several studies have focused on the Fanconi anemia pathway, which responds to DNA cross-linking damage and chromosomal instability through a yet unknown mechanism involving interactions with BRCA1 and RAD51 and recruitment of BRCA2, in regulation of fragile site stability [101]. Fanconi anemia is an autosomal recessive disease associated with an increase in cancer susceptibility, and is most often a result of mutations in FA genes (subtypes A, B, C, D1, D2, E, F, G, I, J, L, M, N) [105]. Chromosomal breaks in blood lymphocytes of FA patients are preferentially located at fragile sites [106], and FANCD2 and FANCI specifically associate with common fragile site loci under conditions of replication stress [107]. Also, ATR phosphorylates the FA protein, FANCD2, and is required for its monoubiquitination [108], which is necessary for its activation during S-phase and subsequent colocalization with BRCA1 and RAD51[109], in response to replication stress. Treatment of both FANCD2 knockdown cells and FA-patient cells with APH results in increased fragile site breakage [101]. Interestingly, cigarette smoke, a known induction agent of fragile site expression, suppresses FANCD2 expression in airway epithelial cells [110]. It will be intriguing to investigate the connection between the cancer susceptibility of FA patients and fragile site-mediated cancer-causing rearrangements.

WRN is an ATP-dependent 3’-5’ helicase and 3’-5’ exonuclease that is targeted by ATR and interacts with ATR-pathway proteins. Increased fragile site breakage is seen in cells of patients with Werner syndrome (a premature aging disease associated with a greater susceptibility to cancer development), and in WRN knockdown cells after treatment with APH [103]. In addition, double knockdown of WRN and ATR did not result in increased chromosomal damage compared to WRN or ATR knockdown alone, suggesting these proteins work in a common pathway. The activity of WRN in fragile site maintenance still remains unclear. Pirzio *et al.* presented data suggesting that WRN helicase, not exonuclease activity, plays the main role in stabilizing fragile sites [103]. In contrast, Shah *et al.* found that neither WRN helicase or exonuclease activity was necessary for polymerase δ progression past stalled replication forks within various FRA16D sequences *in vitro* [106].

While the importance of the ATR pathway in fragile site maintenance has been established, the mechanism is not fully understood. Recently, Wan *et al.* found that ATR binds (directly or through complexes) to fragile site FRA3B preferentially compared to non-fragile regions under conditions of mild replication stress [111]. This binding increases in a dose-dependent manner, peaking at 0.4μM APH, and decreases at higher APH concentrations. While the level of ATR binding to FRA3B changes with treatment, the cellular levels of ATR, phospho-ATR (Ser 428), and ATR interacting proteins ATRIP and TopBP1 remain unchanged. This suggests that ATR binding to the fragile site is guided initially by the level of replication stress signals generated at FRA3B due to APH treatment, and then sequestered from FRA3B regions by successive signals from other non-fragile site regions, which are produced at the higher concentrations of APH. Furthermore, the kinase activity of ATR was required for ATR binding to FRA3B in response to APH treatment. While ATR kinase activity is known to be necessary for phosphorylation of downstream targets to activate the checkpoint signaling cascade [93], these data indicate that the kinase activity of ATR is also necessary for ATR interaction to fragile site regions, most likely through phosphorylation of ATRIP and TopBP1 to stabilize the interaction between these three proteins and the fragile DNA. Two models which are not mutually exclusive have been proposed to explain how ATR helps to maintain fragile site stability [112]. The first model states that a loss of ATR can lead to a bypass of stalled replication forks at fragile sites, ultimately resulting in a failure of checkpoint pathways to prevent entry into mitosis, thus leaving DNA breakage at the unreplicated DNA. The second model states that a loss of ATR leads to replication fork collapse at fragile sites and improper resolution of these structures by ATR leads to DNA breaks. The current information about the involvement of ATR at fragile sites supports a combination of both models. The preferential binding of ATR protein to FRA3B fragile DNAs following APH treatment [111] suggests that ATR plays a possible local role in stabilizing stalled replication forks at fragile regions. Also, this binding and increased fragile site breakage following the inhibition of various members of the ATR pathway suggest that ATR response to fragile sites under conditions of replication stress can activate the ATR-dependent pathway. Finally, decreased ATR binding to FRA3B at higher concentrations of APH [111], which induce more chromosomal gaps or genomic breaks, supports the idea that DNA breakage at fragile sites is due to a failure of ATR to stabilize replication forks and to signal a checkpoint response.

ATR and many downstream proteins (e.g. ATM, BRCA1, CHK1, HUS1, SMC1, FANCD2, WRN, and claspin) are important in maintaining fragile site stability. However, in response to replication stress, whether these proteins act in the ATR pathway and their specific role as adaptor, transducer or effector molecules, and/or participate in an alternative pathway, remains unclear. More research is necessary to better understand the mechanism of the ATR pathway at fragile sites and its effect on cancer development.

## PERSPECTIVES

**Fig. (1):**
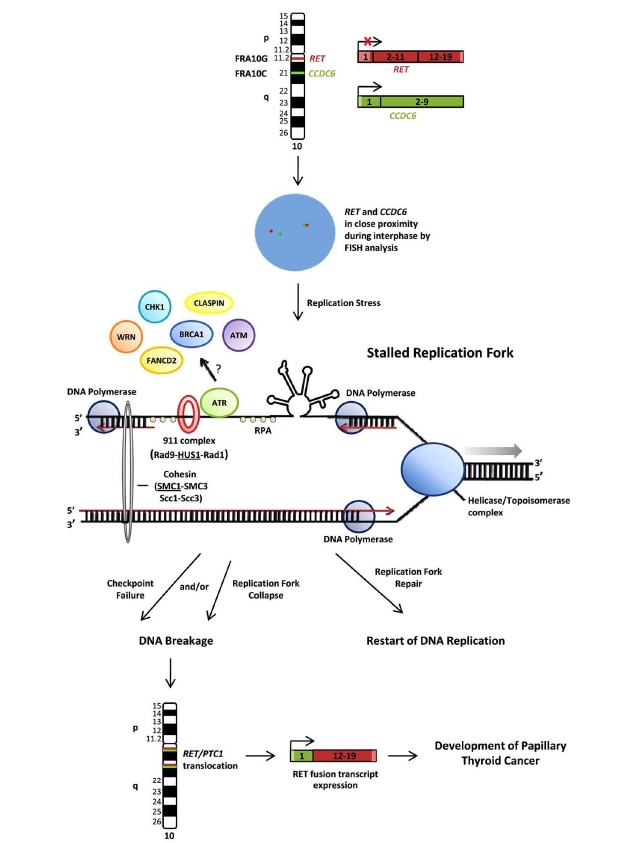
**Model of fragile site instability in the formation of cancer-specific chromosomal rearrangements.** *RET* and *CCDC6* genes, located on chromosome 10 within the fragile sites FRA10G and FRA10C respectively, are closer than expected during interphase in normal thyroid cells. In the normal thyroid gland, *RET* is not expressed in follicular cells, while *CCDC6* is constitutively expressed. Under conditions of replication stress, replicative DNA polymerases α, δ, and ε become uncoupled from the helicase/topoisomerase complex, resulting in long stretches on single-stranded DNA susceptible to the formation of stable secondary structures. These structures can cause replication fork stalling, triggering the ATR-dependent DNA damage checkpoint pathway. Fragile sites may also be susceptible to spontaneous fork reversal or polymerase skipping at regions of secondary structure. For repair of stalled forks, ATR binds to the fragile DNA either directly or through complexes, and activates a downstream signaling cascade with other proteins, including CHK1, BRCA1, FANCD2, WRN, Claspin, HUS1, ATM, and SMC1. If the ATR pathway properly responds, the replication fork will be repaired and DNA replication will resume normally. A loss, deficiency, or defect in ATR pathway proteins could lead to checkpoint failure and/or replication fork collapse resulting in DNA breakage at *RET* and *CCDC6*. DNA breakage at these sites can lead to the formation of *RET/PTC1* translocations, the expression of oncogenic RET protein, and the development of papillary thyroid carcinoma.

Herein we present a model for fragile site instability in the formation of cancer-specific chromosomal rearrangements in the context of *RET/PTC1* rearrangement formation, since fragile site breakage at RET and CCDC6 directly results in the formation of translocations like those observed in patient tumors (Fig.**1**). *RET* and *CCDC6* genes, located within the fragile sites FRA10G and FRA10C respectively, are in close proximity during interphase in normal thyroid cells [72, 73], thus promoting the formation of rearrangement. Under conditions of replication stress, such as those environmental and dietary agents known to induce fragile sites, replicative DNA polymerases become uncoupled from the helicase/topoisomerase complex, resulting in long stretches on single-stranded DNA. Regions that maintain single-strandedness, such as the OIZ, may promote the formation of stable secondary structures due to the intrinsic features of fragile DNA. These structures can cause significant difficulties during replication, resulting in a stalled replication fork. The ATR-dependent DNA damage checkpoint pathway, which responds to stalled or collapsed replication forks, is then triggered. In some cases, a stalled replication fork may spontaneously regress during synthesis of fragile DNA, generating a Holliday junction-like intermediate which could lead to breakage through cleavage by resolvase enzymes [113], or polymerase bypass might occur at regions of structure formation resulting in chromosome deletions. For repair of stalled forks, ATR, the main sensor of the pathway, binds to the fragile DNA either directly or through complexes and activates a downstream signaling cascade through phosphorylation of various targets, including central regulator CHK1. Other components of the ATR pathway, including BRCA1, FANCD2, WRN, Claspin, HUS1, and SMC1, are crucial for fragile site maintenance, although their direct role remains unclear. If the ATR pathway properly responds, the replication fork will be repaired and DNA replication will resume normally. However, a loss, deficiency, or defect in ATR pathway proteins could lead to checkpoint failure and/or replication fork collapse resulting in DNA breakage, the initiating event of translocation formation. Therefore, breakage at *RET* and *CCDC6* can lead to the formation of *RET/PTC1* translocations in sporadic tumors through a fragile site-mediated mechanism, resulting in the development of sporadic papillary thyroid carcinomas.

Several studies have shown a strong correlation between the location of fragile sites and sites of breakage in a few cancer-specific chromosomal aberrations. We showed that over half of breakpoints in all reported recurrent cancer-specific chromosomal translocations are within known fragile sites, but that this is an underestimation, since only simple translocations were investigated [65]. There may be other fragile sites in the genome as yet unidentified. A recent report by Mrasek *et al.* found 61 fragile sites previously not observed and 52 previously not verified, all of which are APH-inducible [14]. Intriguingly, all classes of rare and common fragile sites were induced by APH. These results suggest that fragile sites may have an even greater role in carcinogenesis than previously thought. Since certain dietary and environmental factors can induce or enhance breakage of various APH-induced common fragile sites, it is conceivable that the newly identified sites are also targeted by these agents, thus increasing the risk of cancer development. Futhermore, these results strongly suggest a role for fragile sites in the development of sporadic cancer. Future studies are needed to elucidate the mechanism of fragile site instability in the formation of cancer-specific chromosomal aberrations and the contribution of dietary and environmental factors in this process. 

## Figures and Tables

**Table 1.** Environmental, Dietary and Medicinal Inducers/Enhancers of Fragile Sites

Chemical/Condition	Uses	References
5-azacytidine	chemotherapeutic agent	[40]
actinomycin D	chemotherapeutic agent	[40]
atenolol	hypertension drug	[41]
benzene	found in cigarette smoke, gasoline fumes	[40]
bleomycin	chemotherapeutic agent	[40]
busulfan	chemotherapeutic agent	[40]
caffeine	dietary agent	[5, 29]
carbon tetrachloride	found in refrigerants, pesticides	[40]
chlorambucil	chemotherapeutic agent	[40]
cigarette smoke	dietary and environmental agent	[33, 34]
cytosine arabinoside	chemotherapeutic agent	[40]
diethylnitrosamine	found in cigarette smoke, pesticides, cured meat, whiskey	[40]
dimethyl sulfate	found in dyes, drugs, perfumes, pesticides	[40]
ethanol	dietary agent	[30, 31]
FUdR	chemotherapeutic agent	[40]
hypoxia	low oxygen; found in tumor microenvironment	[32]
methotrexate	chemotherapeutic agent	[40]
pesticides	environmental agent	[35, 36, 39]

**Table 2.** DNA Damage Checkpoint Proteins Shown to Regulate Common Fragile Site Stability

Protein	Function	Reference
ATM	Kinase, maintains fragile site stability in the absense of ATR	[99]
ATR	Kinase, binds to fragile DNA in response to replication stress, phosphorylates downstream targets to activate checkpoint response	[23, 111]
BRCA1	Phosphorylated by ATR, major downstream target of ATR, necessary for G_2_/M checkpoint activation following replication stress	[25]
CHK1	Kinase, phosphorylated by ATR in response to replication stress, central regulator of ATR pathway	[26]
Claspin	Phosphorylated and interacts with CHK1 in response to replication stress	[104]
FANCD2	Fanconi Anemia pathway protein, phosphorylated by ATR leading to activation by mono-Ub, activated by replication stress	[101]
HUS1	Member of the 9-1-1 complex, promotes phosphorylation of ATR substrates	[102]
SMC1	Chromosomal structural maintenance protein, member of the cohesion complex	[100]
WRN	ATP-dependent 3’-5’ helicase, 3’-5’ exonuclease	[91, 103]
